# Supplementary material for: miR-149-3p reverses CD8+ T-cell exhaustion by reducing inhibitory receptors and promoting cytokine secretion in breast cancer cells
Source: Open Biol. 2019 Oct 9;9(10):190061. doi: 10.1098/rsob.190061 (PMC6833224; doi:10.1098/rsob.190061)
Supplement: Table S1. miRNA that are differentially expressed in CD8+PD-1+ vs. CD8+PD-1- T cells [file rsob190061supp1.pdf]

Supplementary Materials and Methods

Gene expression analysis of IL-2, TNF-α, and IFN-γ

A sensiFASTTM Probe NO-ROX Kit (Bioline, USA) was used to conduct RT-qPCR reactions. Primers of murine IL-2, TNF-α, IFN-γ, and internal control gene β-actin were: IL-2: 5'-ACATTGACACTTGTGCTCCGTGTC-3' (forward) and 5'-TTGAGGGCTTGTTGAGATGATGCT-3' (reverse); TNF-α: 5'-GCCTCTTCTCATTCCTGCTTGTGG-3' (forward) and 5'-CCCGTTATCTCCCCTTCATCTTCC-3' (reverse); IFN-γ: 5'-TGAGACAATGAACGCTACAC-3' (forward) and 5'-GGAGCCCTTTAGGCTAGCAG-3' (reverse); β-actin: 5'-AGGGAAATCGTGCGTGACAT-3' (forward) and 5'-CTTCCACATCTATGCCACT-3' (reverse). RT-PCR reaction conditions were: 95° C for 2 min, followed by 40 cycles at 95° C for 10 sec, 58° C for 10 sec, then 95° C for 10 sec. The qPCR results were analyzed using 2-ΔΔCt method [47].

Supplementary Figure Legends

Supplementary Figure 1

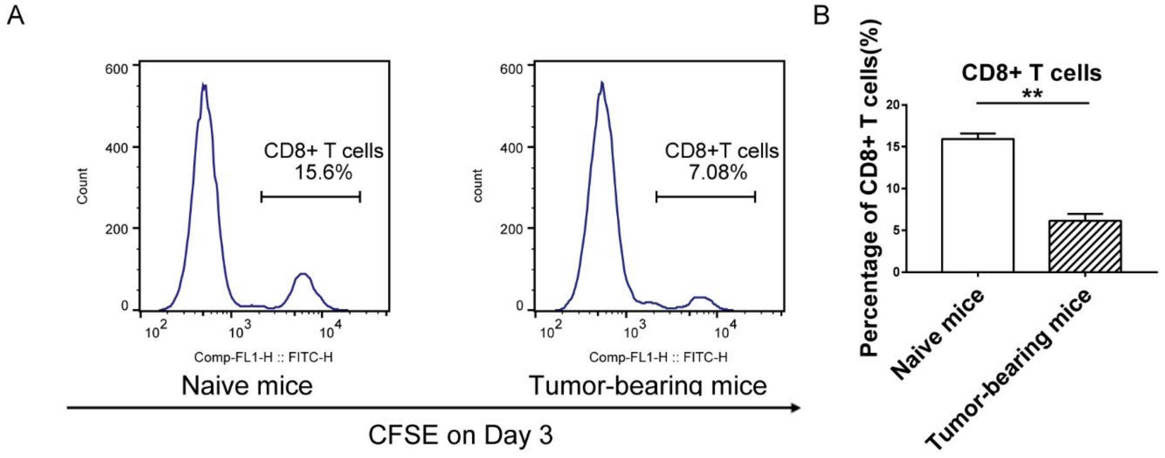

Supplementary Figure S1. Percentages of splenic CD8+ T cells in naive and tumor-bearing mice. Spleen cells were collected from naive mice and tumor-bearing mice on day 18 after tumor cell injection. Flow cytometry was performed after FITC anti-mouse CD8 staining (**A**, B). Data are representative of three independent experiments. Unpaired Student *t*-tests were performed to determine statistical significance (** $p < 0.01$).

Supplementary Figure 2

A

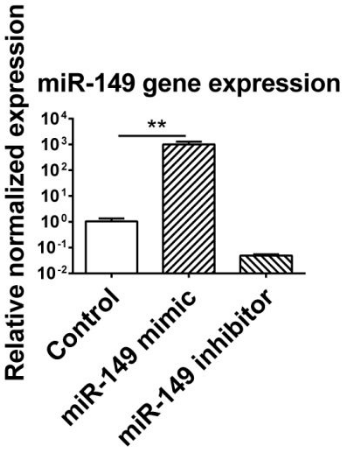

Supplementary Figure S2. Detection of miR-149−3p level after miRNA transfection. CD8+ T cells were purified from the tumor-bearing mice using Miltenyi magnetically-labeled beads and transfected with control miRNA, miR-149-3p mimics, and miR-149-3p inhibitors for 48 hrs. After transfection, miRNeasy Mini Kit and miScript II Reverse Transcriptase Kit were used for miRNA isolation and cDNA synthesis. miR-149−3p levels were detected by RT-qPCR using miScript Primer Assay. Data are representative of three independent experiments. Unpaired Student's t-tests were performed to determine statistical significance (** $p < 0.01$).

Supplementary Figure 3

A

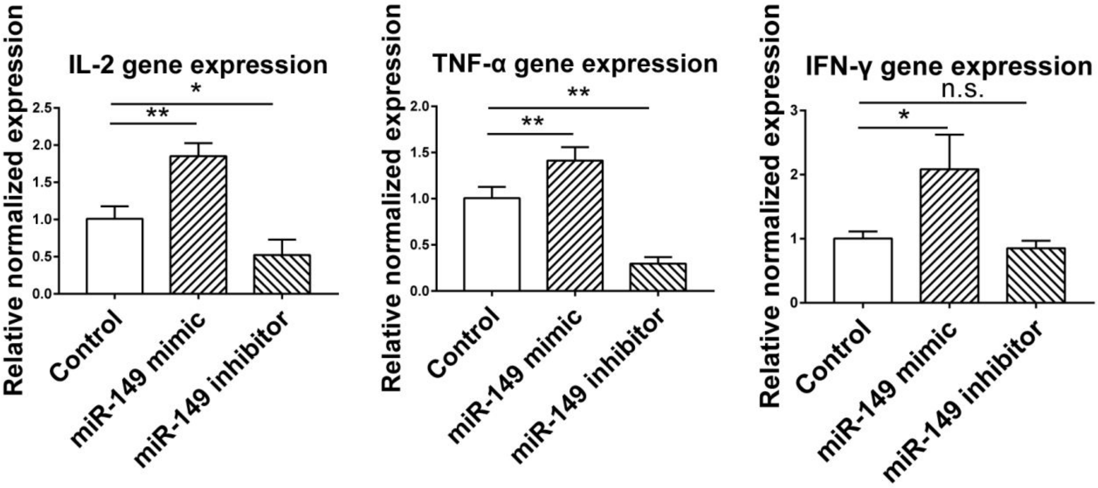

Supplementary Figure S3. miR-149-3p restored activity-associated cytokine levels in exhausted CD8+ T cells. (A) Detection of cytokine IL-2, TNF-α and IFN-γ levels by RT-qPCR. Spleen cells collected from tumor-bearing mice were respectively transfected with control miRNA, miR-149-3p mimics and miR-149-3p inhibitors for 48 hrs. After transfection, CD8+ T cells were purified from the collected splenocytes using Miltenyi magnetically-labeled beads. Detection of cytokine IL-2, TNF-α, and IFN-γ levels on CD8+ T cells by qPCR were performed using CD8+ T cells mRNA. Data are representative of three independent experiments. Unpaired Student's t-tests were performed to determine statistical significance (* $p < 0.05$, ** $p < 0.01$).

Supplementary Table 1. miRNA that are differential expressed in CD8+PD-1+ vs. CD8+PD-1- T cells

miRNA	Fold Change	P value
mmu-miR-877-3p	-4.63246	0.0481926
mmu-miR-122-5p	-7.04076	0.00461435
mmu-miR-182-5p	-6.84349	0.00217941
mmu-miR-31-5p	-7.74298	0.000429221
mmu-miR-183-5p	-4.86247	0.0064926
mmu-miR-493-3p	-4.19264	0.0127347
mmu-miR-146b-5p	-5.57134	0.0016485
mmu-miR-2182	-3.64419	0.0181424
mmu-miR-146a-5p	-7.13698	1.56E-05
mmu-miR-132-3p	-4.45666	0.00360028
mmu-miR-451a	-3.88719	0.0072596
mmu-miR-3100-5p	-3.89221	0.00588115
mmu-miR-3090-5p	-3.03999	0.0314833
mmu-miR-301a-3p	-4.46581	0.00124239
mmu-miR-721	-2.98771	0.0285459
mmu-miR-1897-5p	-2.94036	0.0309768
mmu-miR-31-3p	-3.47111	0.00803287
mmu-miR-1249-5p	-3.13886	0.014691
mmu-miR-1946b	-2.7138	0.038924
mmu-miR-5133	-2.70771	0.0275184
mmu-miR-3107	-2.48916	0.0428741
mmu-miR-208a-5p	-4.61288	5.23E-06
mmu-miR-155-5p	-4.37146	1.47E-05
mmu-miR-503-5p	-3.21989	0.00124764
mmu-miR-770-3p	-2.23397	0.0381449
mmu-miR-1188-5p	-2.18363	0.0440008
mmu-miR-1892	-2.33103	0.0215127
mmu-miR-148a-3p	-2.20839	0.027217
mmu-miR-22-3p	-2.4741	0.00771092
mmu-mir-122-5p	-2.34245	0.0134414
mmu-miR-705	-2.17581	0.0248352
mmu-miR-762	-2.50612	0.00406305
mmu-miR-301b-3p	-2.65939	0.00168337
mmu-miR-323-5p	-2.05377	0.0194033
mmu-miR-2137	-2.09987	0.0127005
mmu-miR-1982-5p	-2.48098	0.00121774
mmu-miR-3113-3p	-1.90555	0.0346454
mmu-miR-486a-5p	-1.88165	0.0387998
mmu-miR-711	-2.25475	0.00422179
mmu-miR-16-1-3p	-1.86254	0.0369852
mmu-miR-683	-1.85419	0.0380149
mmu-miR-328-5p	-1.93832	0.022855
mmu-miR-667-3p	-2.13814	0.00525438
mmu-miR-760-3p	-1.97621	0.0136702
mmu-miR-21-5p	-2.19774	0.00265697
mmu-miR-1306-3p	-1.76458	0.044537
mmu-miR-152-3p	-1.99323	0.00625333
mmu-miR-2861	-2.07494	0.00201641

mmu-miR-135a-1-3p	-1.7819	0.0178396
mmu-miR-3077-5p	-1.97432	0.00307048
mmu-miR-1956	-1.78871	0.0125691
mmu-miR-3092-3p	-1.67789	0.0272706
mmu-miR-3103-5p	-1.65124	0.0311412
mmu-miR-3473b	-1.69738	0.0199922
mmu-miR-211-3p	-1.58515	0.0459556
mmu-miR-338-5p	-1.59045	0.0411785
mmu-miR-5120	-1.58318	0.0389385
mmu-miR-486a-3p	-1.63727	0.0193264
mmu-miR-3473b	-1.83411	0.00209965
mmu-miR-3073-3p	-1.61321	0.0181186
mmu-miR-5112	-1.53973	0.0361025
mmu-miR-692-1	-1.67981	0.00788799
mmu-miR-5122	-1.65328	0.0100303
mmu-miR-149-3p	-1.70489	0.00502392
mmu-miR-5126	-1.80542	0.00136424
mmu-mir-181a-5p	-1.62387	0.0100198
mmu-miR-199b-3p	-1.79818	0.000266562
mmu-miR-3960	-1.69573	0.00150044
mmu-miR-329-3p	-1.5802	0.00692929
mmu-miR-495-3p	-1.54442	0.00847919
mmu-miR-671-5p	-1.61961	0.000532439
mmu-miR-195-5p	1.74101	0.049403
mmu-miR-7a-5p	1.59813	0.0427475
mmu-miR-592-5p	1.68996	0.0427483
mmu-miR-141-3p	1.50047	0.0328156
mmu-miR-713	1.69498	0.0362003
mmu-miR-710	1.57297	0.0334074
mmu-miR-30a-5p	1.72269	0.0307059
mmu-miR-674-5p	1.63209	0.0281633
mmu-miR-532-5p	1.74271	0.0292923
mmu-miR-200b-3p	2.16848	0.0333822
mmu-miR-26b-5p	1.73628	0.0214963
mmu-miR-466c-3p	1.51466	0.00872042
mmu-miR-101a-5p	1.52329	0.00838995
mmu-miR-17-3p	1.50002	0.00687202
mmu-miR-466e-3p	1.62942	0.0107894
mmu-miR-466b-3p	1.52605	0.0074928
mmu-miR-29c-3p	1.76927	0.0142901
mmu-miR-466a-3p	1.70988	0.0122977
mmu-miR-320-3p	1.59862	0.00844204
mmu-miR-140-3p	1.52482	0.00383394
mmu-miR-361-5p	1.56647	0.00352102
mmu-miR-466p-3p	1.64667	0.00500277
mmu-miR-3096-5p	2.13493	0.0136412
mmu-miR-7a-1-3p	1.85244	0.00810395
mmu-miR-342-5p	1.8386	0.00478512
mmu-miR-203-3p	2.25974	0.0106265
mmu-miR-1839-5p	1.70537	0.00224937

mmu-miR-361-5p	1.59818	0.000822977
mmu-miR-30b-5p	1.69095	0.00110493
mmu-miR-30d-5p	1.90078	0.00284284
mmu-miR-200a-3p	3.64187	0.0172061
mmu-miR-192-5p	1.78536	0.000934273
mmu-miR-101b-3p	2.21944	0.00469913
mmu-miR-3096b	2.45362	0.00633874
mmu-miR-467e-5p	1.67609	9.49E-05
mmu-miR-194-5p	1.9913	0.00172014
mmu-miR-151-3p	3.69674	0.0132786
mmu-miR-28-3p	2.30235	0.00328795
mmu-miR-29b-3p	2.33633	0.00306258
mmu-miR-378a-3p	2.12837	0.00126477
mmu-miR-5121	2.07712	0.000632864
mmu-miR-378b	2.06945	0.000207061
mmu-miR-3096-3p	3.12294	0.00369122
mmu-miR-139-5p	2.77285	0.00103898
mmu-miR-3096b-3p	4.00412	0.00490373
mmu-miR-151-5p	3.85989	0.00069181
